# Loin Pain Hematuria Syndrome—a case report

**DOI:** 10.1093/jscr/rjab246

**Published:** 2021-11-11

**Authors:** Ehtisham Zeb, Adam O'Connor, Shariq Sabri, Karim Muhammad, Nafees Qureshi

**Affiliations:** Department of General Surgery, Tameside and Glossop Integrated Care NHS Foundation Trust, Ashton-Under-Lyne, United Kingdom; Department of General Surgery, Tameside and Glossop Integrated Care NHS Foundation Trust, Ashton-Under-Lyne, United Kingdom; Department of General Surgery, Tameside and Glossop Integrated Care NHS Foundation Trust, Ashton-Under-Lyne, United Kingdom; Department of General Surgery, Tameside and Glossop Integrated Care NHS Foundation Trust, Ashton-Under-Lyne, United Kingdom; Department of General Surgery, Tameside and Glossop Integrated Care NHS Foundation Trust, Ashton-Under-Lyne, United Kingdom

**Keywords:** LPHS, autotransplant kidney, recurrent UTI, chronic inflammation, pain management, somatization disorder, rooftop incision

## Abstract

Loin pain hematuria syndrome (LPHS) is a rare idiopathic condition. LPHS can present with both unilateral and bilateral loin pain, microscopic or macroscopic hematuria. It is a diagnosis of exclusion. The management options for this condition include pain management with narcotics or opioids, renal denervation, kidney autotransplantation and neurectomy or nephrectomy. However, these treatment modalities are the last resort.

## INTRODUCTION

Loin Pain Hematuria Syndrome (LPHS) was first described in 1967 by Little *et al* [1]. The most common presentation of LPHS is severe unilateral or bilateral flank pain, which radiates to the abdomen, medial aspects of the thigh or groin region. The pain in LPHS can be severe and excruciating mimicking acute renal pain and has been described to be one of the worst pains experienced by patients. This pain has been documented in the past to be exacerbated by daily routine activities, such as lifting weights, driving a car, operating a machinery or exercise. LPHS is though considered as a diagnosis of exclusion. Due to severe pain experienced in LPHS, patients usually require high doses of narcotic analgesics to obtain optimal pain control. Additionally, patients have reported microscopic and macroscopic hematuria along the course of their disease. As LPHS is a debilitating condition affecting the day-to-day life of patients, it can also have an impact on the general physical and mental health of patients. Along with symptom/pain control management, some interventional treatments have also been established to manage LPHS, including renal denervation procedures, kidney autotransplantation and ultimately a unilateral nephrectomy, though with variable success rates.

## CASE REPORT

**Figure 1:**
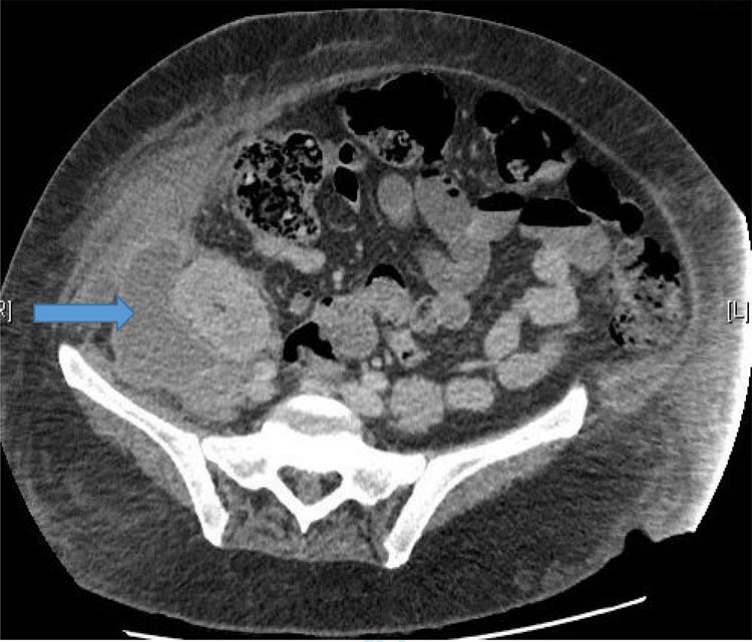
Large right retroperitoneal loculated fluid collection superior to the right pelvic kidney with no internal gaseous contents.

**Figure 2:**
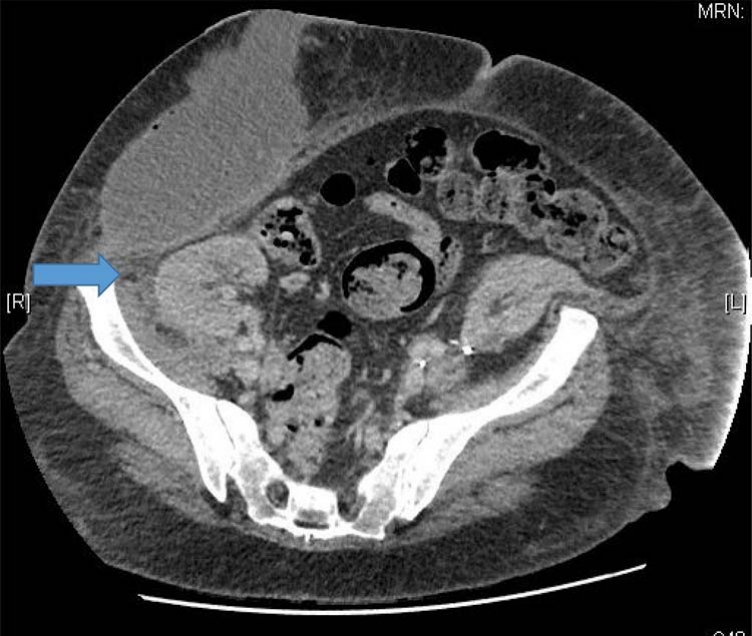
Tiny gas pockets noted close to the anterior margin.

A 48-year-old lady was admitted from the outpatient surgical clinic with a painful lump in the right iliac fossa for a duration of 4 weeks. The patient had undergone kidney autotransplantation long time ago and she had a rooftop incision over the overlying kidneys. Patient’s initial diagnostic impression was a query painful obstructed incisional hernia as a result of the previous renal surgery to denervate the nerves and re-implant the kidneys to a different location within the abdominal cavity. In order to confirm the diagnosis, a computed tomography (CT)-abdomen and pelvis with IV contrast was organized. The scan showed a large retroperitoneal loculated collection with tracking of fluid into the anterior abdominal wall, where there were tiny gas pockets close to the anterior margin (Figs 1 and 2). The patient was transfused with three units of packed red cells, given the impression of a chronic hematoma as per the CT scan. Our patient had undergone bilateral autotransplanted kidneys in the iliac fossa region.

The patient was optimized with blood transfusion prior to the procedure.

## DISCUSSION

LPHS is a rare condition which has not been completely understood by scientists and clinicians but has been reported by clinicians across the world. A variety of conditions are often on the list of differential diagnosis for patients with LPHS. These differentials include ureteric obstruction, malignancy, recurrent thromboembolism and nephropathies, like Immunoglobulin A (IgA) nephropathy, arteriovenous fistulas or nut cracker syndrome, causing compression of the left renal vein between the aorta and superior mesenteric artery. Our patient had undergone stripping of the renal capsule from the kidney capsulotomy and stripping of the renal nerves from the renal artery (neurectomy).

LPHS describes a constellation of symptoms that is estimated to have a prevalence of ~0.012% and primarily occurs in women [2]. The most prominent clinical features include periods of severe intermittent or persistent unilateral or bilateral loin pain accompanied by either microscopic or gross hematuria [3]. Kidney biopsies from patients with loin pain hematuria typically reveal only minor pathologic abnormalities. LPHS has previously been differentiated as Type 1 or Type 2 LPHS. Type 1 LPHS can be attributed to identifiable causes, including nutcracker syndrome, nephrolithiasis, polycystic kidney disease, recurrent renal papillary necrosis with ureteral obstruction, renal thromboembolism or renal artery dissection. Cases in which diagnostic work-up does not reveal an etiology have been categorized as Type 2 LPHS [4]. Further, LPHS is not associated with the loss of kidney function or urinary tract infections. It has also been concluded from research that LPHS-associated hematuria and the pain it causes can be related to the vascular disease of the kidney, coagulopathy, renal vasospasm with microinfarction, hypersensitivity, complement activation on arterioles, veno-calyceal fistula, abnormal ureteral peristalsis and intra-tubular deposition of calcium or uric acid microcrystals. The natural history of the syndrome is not well described, but spontaneous resolution has been suggested. LPHS is not known to cause secondary kidney injury or increase mortality.

In a series of 51 patients who were studied and followed up, LPHS resolved spontaneously in half of the patients after several years (mean: 3–5); the rest of the patients continued to have pain with the longest duration of pain documented for 17 years [5]. However, the lack of conclusive pathophysiology, in conjunction with the rarity of LPHS, has prohibited scientists in the development and trial of definitive treatment options [6]. Renal denervation cures severe intractable pain in about 25% of patients. Recurrence of pain could be prevented in more patients if there was a way of preventing re-innervation [7]. After autotransplantation, most patients are weaned off of narcotics and can go back to their daily routines within a few weeks. LPHS continues to be not only a rare disease but it is also incompletely understood. Nevertheless, patients who are affected by it experience life-altering pain, which frequently leads to disability. Although conservative management and surgical intervention have varied results, multiple case series have reported high success rates with renal autotransplant.

It has been reported in Vakili and associates that Burke and Chin presented a series of 48 patients who underwent renal autotransplantation with sustained pain relief in 70% of these patients [8, 9]. However, smaller series have reported pain recurrence up to 73% [10, 11]. Researchers believe one key explanation to this discrepancy in outcomes is patient selection. Since research has identified the ureter as a contributor to pain through ureteral spasm, researchers have developed the UW-LPHS test as a method to identify those patients who may benefit from renal autotransplantation. Although additional follow-up is required, the current results indicate that it is a reliable method to identify these patients. As a result, researchers have supported the UW-LPHS test as a simple and reliable diagnostic tool in order to help determine the best treatment for patients with LPHS.

## CONCLUSION

The awareness of LPHS is very limited among physicians and surgeons. It has been associated mistakenly with psychiatric disorders, such as somatization disorders. Our patient, despite having autotransplanted kidneys 8 years ago and being asymptomatic for this duration, developed a large intra-abdominal collection adjacent to the transplanted kidney which was communicating with the abdominal wall; and the patient ended up with an emergency operation to drain the hematoma and a corrugated drain placed *in situ*, which was followed by serial CT scans to manage the underlying cause. There were no issues in her long-term follow-up.

## CONFLICT OF INTEREST STATEMENT

None declared.

## References

[1] Little PJ, Sloper JS, de Wardener HE. A syndrome of loin pain and haematuria associated with disease of peripheral renal arteries. Q J Med 1967;36:253–9. 4227314

[2] Eisenberg ML , Lee KL, Zumrutbas AE, Meng MV, Freise CE, Stoller ML. Long-term outcomes and late complications of laparoscopic nephrectomy with renal autotransplantation. J Urol 2008;179:240–3. 18001789 10.1016/j.juro.2007.08.135

[3] Taba Taba Vakili S , Alam T, Sollinger H. Loin pain hematuria syndrome. Am J Kidney Dis 2014;64:460–72. 24725981 10.1053/j.ajkd.2014.01.439

[4] Sollinger HW , Al-Qaoud T, Bath N, Redfield RR. The "UW-LPHS Test": a new test to predict the outcome of renal autotransplant for loin pain hematuria syndrome. Exp Clin Transplant 2018;16:651–5. 10.6002/ect.2018.0236 PMC6478157 30251941

[5] Sheil AG , Ibels LS, Thomas MA, Graham JC. Renal autotransplantation for severe loin-pain/haematuria syndrome. Lancet 1985;2:1216–7 2866294 10.1016/s0140-6736(85)90744-5

[6] Urits I , Li N, Berger AA, Walker P, Wesp B, Zamarripa AM, et al. Treatment and management of loin pain hematuria syndrome. Curr Pain Headache Rep 2021;25:6. 33495883 10.1007/s11916-020-00925-0

[7] Andrews BT , Jones NF, Browse NL, Andrews BT. The use of surgical sympathectomy in the treatment of chronic renal pain. Br J Urol 1997;80:6–10. 9240172 10.1046/j.1464-410x.1997.00231.x

[8] Chin JL , Kloth D, Pautler SE, Mulligan M. Renal autotransplantation for the loin pain-hematuria syndrome: long-term followup of 26 cases. J Urol 1998;160:1232–5 discussion 1235-1236. 9751325

[9] Burke JR , Hardie IR. Loin Pain Haematuria Syndrome. Pediatric Nephrology (Berlin, Germany) 1996;10:216–20. 10.1007/BF00862087 8703718

[10] Harney J , Rodgers E, Campbell E, Hickey DP. Loin pain-hematuria syndrome: how effective is renal autotransplantation in its treatment. Urology 1994;44:493–6. 7941188 10.1016/s0090-4295(94)80045-6

[11] Parnham AP , Low A, Finch P, Perlman D, Thomas MAB. Recurrent graft pain following renal autotransplantation for Loin Pain Haematuria Syndrome. Br J Urol 1996;78:25–8. 8795395 10.1046/j.1464-410x.1996.00455.x

